# Neurogenesis from Sox2 expressing cells in the adult cerebellar cortex

**DOI:** 10.1038/s41598-017-06150-x

**Published:** 2017-07-21

**Authors:** Julia Ahlfeld, Severin Filser, Felix Schmidt, Annika K. Wefers, Daniel J. Merk, Rainer Glaß, Jochen Herms, Ulrich Schüller

**Affiliations:** 1 Center for Neuropathology, Ludwig-Maximilians-University, Munich, Germany; 2 Division of Clinical Pharmacology, Department of Internal Medicine IV, Ludwig-Maximilians-University, Munich, Germany; 3 German Center for Neurodegenerative Diseases (DZNE), Department for Translational Brain Research, Munich, Germany; 4 Munich Cluster of Systems Neurology (SyNergy), Ludwig-Maximilians-University, Munich, Germany; 5 Division of Neuropathology, Heidelberg University Hospital, Heidelberg, Germany; 6 Cancer Biology and Pediatric Oncology, Dana-Farber Cancer Institute, Boston, Massachusetts USA; 7 Neurobiology, Harvard Medical School, Boston, Massachusetts USA; 8 Department of Neurosurgery, Ludwig-Maximilians-University, Munich, Germany; 9 Institute of Neuropathology, University Medical Center, Hamburg-Eppendorf, Germany; 10 Research Institute Children’s Cancer Center, Hamburg, Germany; 11 Department of Pediatric Hematology and Oncology, University Medical Center, Hamburg-Eppendorf, Germany

## Abstract

We identified a rare undifferentiated cell population that is intermingled with the Bergmann glia of the adult murine cerebellar cortex, expresses the stem cell markers Sox2 and Nestin, and lacks markers of glial or neuronal differentiation. Interestingly, such Sox2^+^ S100^−^ cells of the adult cerebellum expanded after adequate physiological stimuli in mice (exercise), and Sox2^+^ precursors acquired positivity for the neuronal marker NeuN over time and integrated into cellular networks. In human patients, SOX2^+^ S100^−^ cells similarly increased in number after relevant pathological insults (infarcts), suggesting a similar expansion of cells that lack terminal glial differentiation.

## Introduction

Neurogenesis within the adult mammalian brain is traditionally said to be restricted to a few discrete niches^1^, many of which are known to bear precursor cells that express the transcription factor Sox2^2, 3^. Attempts have been made to identify stem cell populations within the adult cerebellum. It has been shown that there is a distinct population of cells within the adult cerebellum that express stem cell markers and that can give rise to neuronal progeny when expanded *in vitro* and subsequently transplanted back into the murine cerebellum^4, 5^.

## Results and Discussion

**Figure 1:**
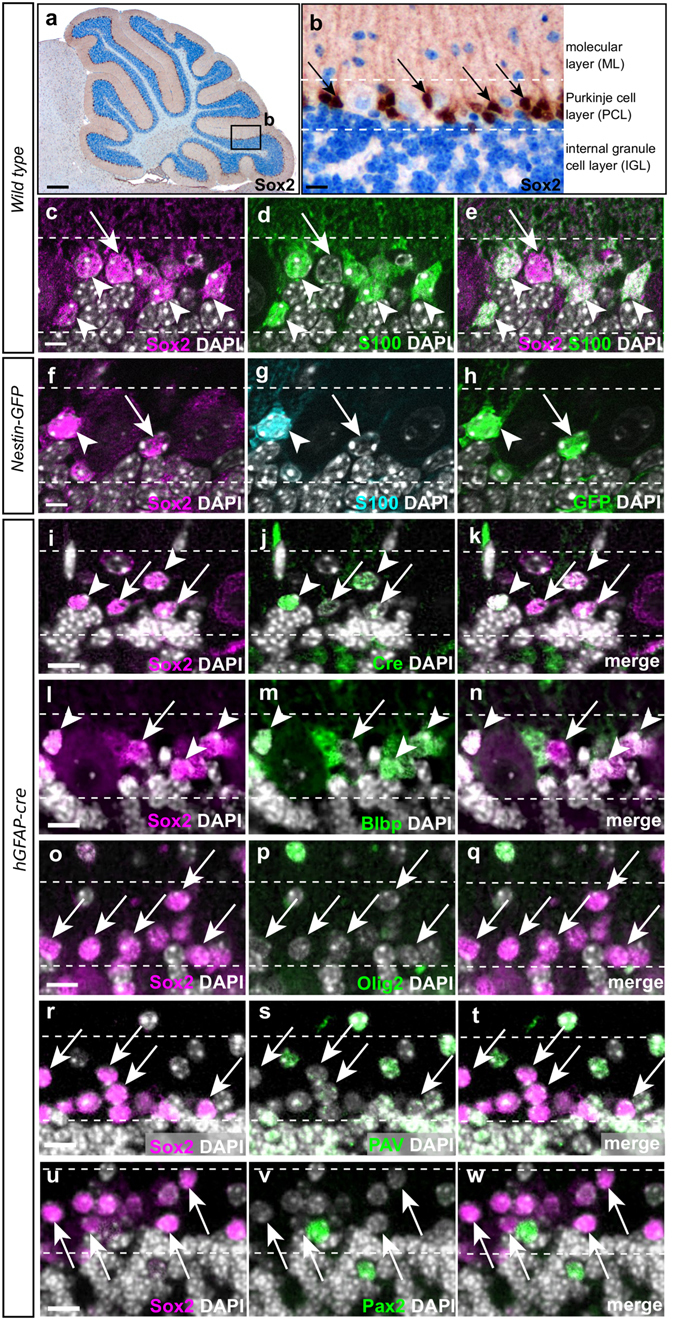
Presence and expansion of undifferentiated Sox2^+^ cells in the cortex of the adult murine cerebellum. Within the adult cerebellar cortex, Sox2 is expressed in small cells within the Purkinje cell layer (PCL) that were believed to represent the Bergmann glia (**a** and arrows in **b**). While most of the Sox2 expressing cells within the PCL stained positive for the glial marker S100B (arrowheads in **c–h**), some of them exclusively expressed Sox2 (arrows in **c–e**). This population was further characterized by Nestin expression, as detected by Nestin-dependent GFP expression (arrows in **f–h**). Cre as nuclear surrogate for GFAP (arrowheads in **i–k**) and Blbp (arrowheads in **l–n**) mark Bergmann glia cells, but Sox2^+^  cells were consistently found that do not co-label with another (single) marker, of differentiation (arrows in **i–n**). Characterization of Sox2-expressing cells within the PCL was done by additional staining for specific cerebellar cell types. Sox2^+^ cells do not express markers of oligodendrocyte lineage (Olig2, arrows in **o–q**) or cerebellar interneurons (Parvalbumin, arrows in **r–t** and Pax2, arrows in **u–w**). Imunohistochemical stainings were performed on 4 µm FFPE sections. White lines show perimeter of Purkinje cell layer. Scale bar in panel a equates to 500 µm, bar in panel b to 50 µm. Scale bars in panels c - w equate to 15 μm.

**Figure 2:**
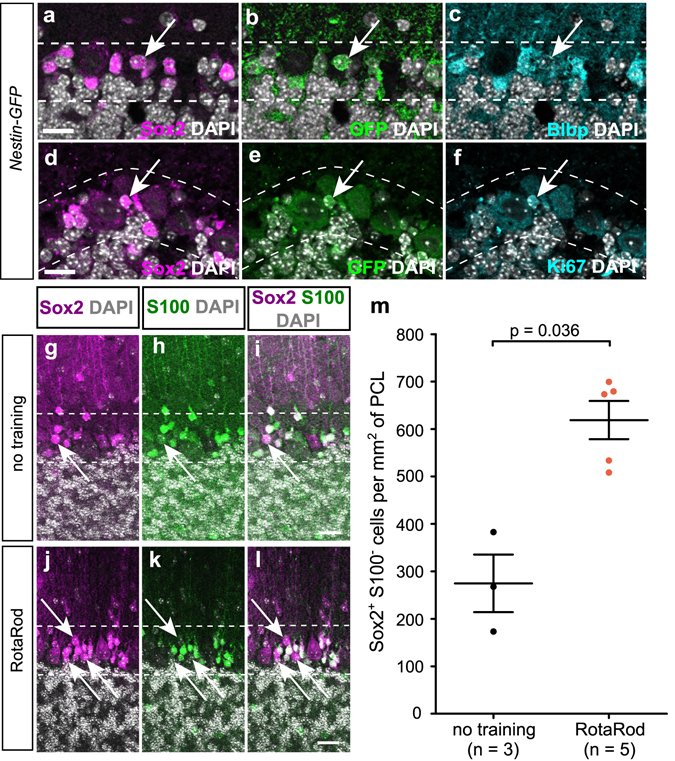
Undifferentiated Sox2^+^ cells express stemness marker Nestin and show reactive properties. Undifferentiated Sox2^+^ cells are characterized by Nestin expression, detected by Nestin-dependent GFP expression and the lack of Blbp expression (**a–c**). Additionally, Sox2^+^ Nestin^+^ cells show proliferative capacity as shown by Ki67 labeling (**d–f**). Sox2^+^ S100^−^ cells were sparsely distributed in the PCL of control animals (**g–i**) when compared to animals that received a week of physical training (arrows in **j–l**). Training of mice on the RotaRod system lead to a significant increase in the number of Sox2^+^ S100^−^ cells within the PCL as compared to controls (**m** – Mann-Whitney-U test; SEM). Immunohistochemical staining in panels a – f was performed on 4 µm FFPE sections. Staining shown in panels g – l was performed on 50 µm free-floating vibratome sections. White lines show perimeter of Purkinje cell layer. Scale bars equate to 50 µm in panels a – f and 100 µm in panels g – l.

**Figure 3:**
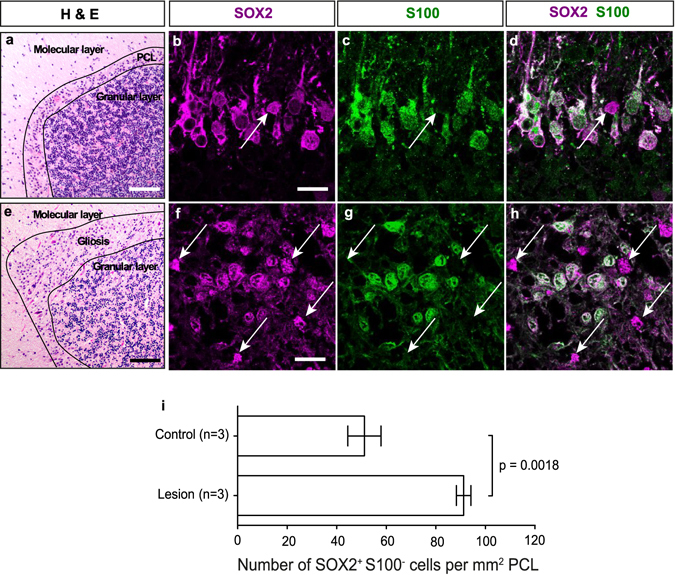
Reactivity of SOX2^+^ S100^−^ cells in the human cerebellum. In human cerebellar tissue (**a**) we found SOX2^+^ S100^−^ cells intermingled with the Bergmann glia of the Purkinje cell layer (arrows in **b–d**). Upon damage to the cerebellum by cerebellar infarcts, we observed reactive gliosis with massive expansion of the Bergmann glia (**e**) as well as expansion of SOX2^+^ S100^−^ cells (**f–i**, standard t-testing; SEM). Immunohistochemical staining was performed on 4 µm FFPE sections. Scale bars in panels a and e equate to 200 µm, scale bars for panels b – h equate to 50 µm.

To identify potential stem cell niches within the adult cerebellum, we carefully investigated the expression pattern of Sox2. As previously described, we found Sox2 expression in a variety of differentiated glia cells, such as Bergmann glia cells that are intermingled with the Purkinje cells^6^ (Fig. 1a and b). However, apart from Bergmann glia cells that are characterized by the co-expression of differentiation markers like S100B, Blbp and Gfap, we found a subset of Sox2-expressing cells in the Purkinje cell layer (PCL) that lack expression of S100, i.e. Sox2^+^ S100^−^ cells that makes up 18.4% of the entire Sox2^+^ population within the PCL (Fig. 1c–e). We further found Sox2^+^ cells that co-express Nestin as shown by Nestin-dependent GFP expression (Fig. 1f–h). To validate our findings, we checked whether there are other markers of cellular differentiation that would label 100% of Bergmann glia cells and consistently found cells that lack expression of Gfap, Blbp, Olig2, Parvalbumin, and Pax2 (Fig. 1i–w). We speculate that there may be a Sox2^+^ population that expresses no other marker of cellular differentiation, but further stainings of multiple markers will be necessary to prove this finding. Finally, Sox2 is absent from any granule cells that are clearly marked by NeuN^7^ (Supplementary Figure 1). Triple labelling revealed that 61.4% of Sox2^+^ Blbp^−^ cells express the stem cell marker Nestin, (Fig. 2a–c; quantification on n = 4 animals; at least 5 sections per cerebellum) and that Sox2^+^ Nestin^+^ cells also exhibit proliferative activity (Fig. 2d–f). When exposing mice to a physical activity paradigm that is commonly used to determine motor defects in research of Parkinson’s disease (RotaRod), we observed a more than 2-fold increase in the number of Sox2^+^ S100^−^ cells within the PCL (Fig. 2j–l), as compared to untrained mice (p = 0.036; Fig. 2g–i, m). This population may therefore have reactive properties that can be triggered by physical exercise. Such SOX2^+^ S100^−^ cells are similarly detectable in the human cerebellum (Fig. 3a–d). Notably, perilesional areas in patients, who suffered from a cerebellar infarct, contained significantly more SOX2^+^ S100^−^ cells than the PCL of control tissue (91.7 cells/mm^2^ (31.1%; SOX2^+^ S100^−^ cells/total SOX2^+^ cells) vs. 49.2 cells/mm^2^ (16.3%; SOX2^+^ S100^−^ cells/total SOX2^+^ cells); p = 0.0018; Fig. 3e–i), while healthy regions adjacent to infarcts maintained the number of SOX2^+^ S100^−^ cells that was also found in healthy control cerebella (49.5 cells/mm^2^ (17.1%; SOX2^+^ S100^−^ cells/total SOX2^+^ cells)), suggesting that reactive properties of these cells may as well be triggered by pathological events.

**Figure 4:**
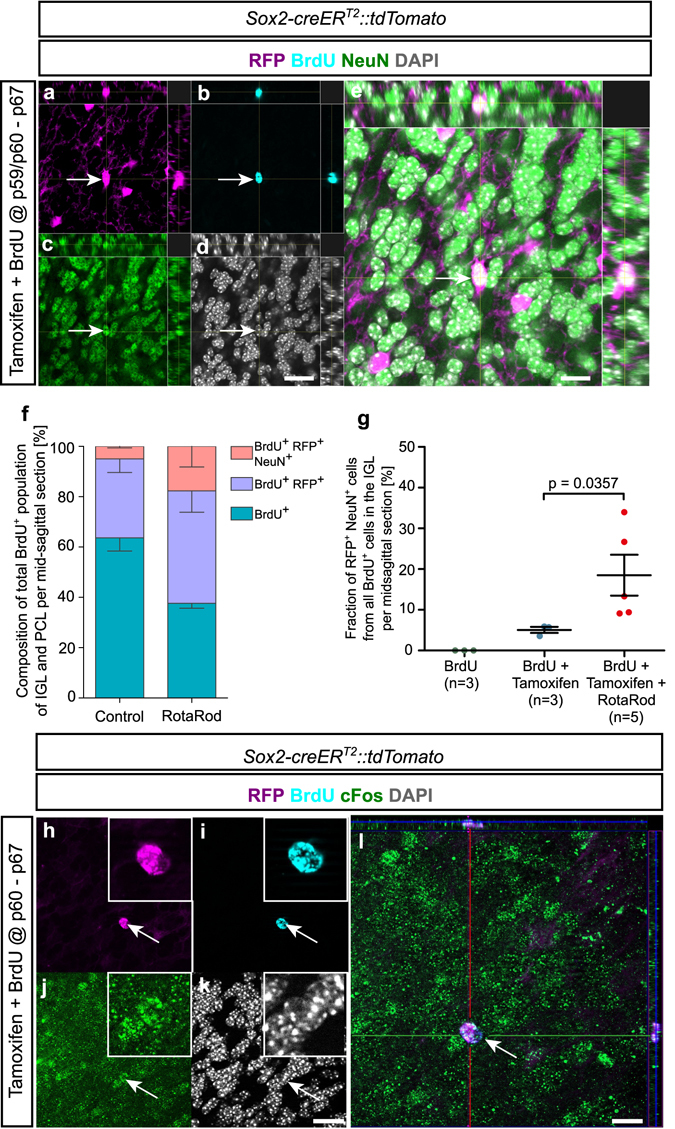
Neurogenesis from Sox2^+^ cells in the adult cerebellar cortex. Combined BrdU birth-dating and fate-mapping experiments show RFP^+^ progeny from Sox2^+^ cells (**a**) that had incorporated BrdU during the observation period (**b**) and had also acquired neuronal differentiation as shown by staining for the granule cell marker NeuN (**c**). Nuclear DAPI staining is shown as a reference in panel d. A maximum intensity projection is shown in panel e with orthogonal views of x-z and y-z planes. The images have been taken after a treatment course with Tamoxifen and BrdU according to the scheme in Supplementary Figure 2. An increase in neurogenesis from Sox2^+^ cells could be achieved by physical activity. Mice that were trained on the RotaRod showed significantly more new-born granule neurons in the internal granule cell layer (IGL), when compared to control animals (**f,g** – Mann-Whitney-U test; SEM). RotaRod data were obtained after a Tamoxifen/BrdU treatment according to the scheme in Supplementary Figure 2. Possible integration of progeny from Sox2^+^ progenitors is shown by positive staining for the activity regulated transcription factor cFos (**h–l**). Immunohistochemical staining was performed on 50 µm free-floating vibratome sections. Scale bars for panels a – d and h – k equate to 40 µm, scale bars in panels e and l equate to 10 µm.

To examine whether Sox2^+^ cells produce any progeny at all within the adult cerebellum under physiological conditions, we injected 2-month-old *Sox2-creER* ^*T2*^ *::tdTomato* mice with Tamoxifen for 8 days and BrdU for the final overlapping 7 days and sacrificed them 5 days after the last injections. The Tamoxifen course was started one day prior to the BrdU course because of the different kinetics of the two systems. BrdU will be detectable in proliferating cells within a few hours after injection. Tamoxifen however, has to be taken up by the cell, dimerized and translocated to the nucleus, where transcription and translation of RFP have to be initiated. Subsequent triple labelling with antibodies against RFP for lineage tracing, BrdU for proliferation since the day of injection and NeuN for neuronal differentiation revealed newly generated cells that originate from Sox2^+^ precursors in the adult cerebellum and express NeuN as a marker of granule neuron differentiation^7^ (Fig. 4a–e). We controlled for unspecific RFP expression by analysing *Sox2-creER* ^*T2*^ *::tdTomato* mice that received BrdU without Tamoxifen injections. By using BrdU-expression as an inclusion criterion, we made sure to include only RFP and NeuN-positive cells that had undergone proliferation since injection, thus excluding any RFP-positive neurons that may have been there since early development. Intriguingly, cerebellar neurogenesis was significantly enhanced by physical activity. The composition of the BrdU^+^ population within PCL and IGL of the adult mouse cerebellum is shown in Fig. 4f. We counted an average of 30 BrdU^+^ cells per mid-sagittal section. While there is no significant difference in the total BrdU^+^ population when comparing control and RotaRod mice, the BrdU^+^ population originating from Sox2 expressing cells (BrdU^+^ RFP^+^) and the BrdU^+^ population originating from Sox2 expressing cells that have differentiated into neurons (BrdU^+^ RFP^+^ NeuN^+^) increased significantly when comparing RotaRod to control mice. Mice that received physical training on a RotaRod also displayed a more than 3-fold increase in RFP^+^ NeuN^+^ cells from all BrdU^+^ cells in midsagittal cerebellar sections (p = 0.0357; Fig. 4g). It has been shown that physical activity can increase the rate, at which new neurons are generated from Sox2^+^ cells within the mature hippocampus ^2^. A similar trend could be observed after equivalent exposure to an enriched environment, where we also found an increase in BrdU^+^ RFP^+^ cells (31.4% control, 45.5% EE; p = 0.2286) and in BrdU^+^ RFP^+^ NeuN^+^ cells (5.1% control, 15.3% EE; p = 0.0571).

Finally, we detected a very small number of RFP^+^ BrdU^+^ cells within the internal granule cell layer of the cerebellum that expressed *cFos*, an immediate early gene that will result in production of an activity-dependent protein^8^, providing first evidence that cells that have been generated by Sox2 expressing progenitors may become integrated into the cellular network of the cerebellum (Fig. 4h–i). At this point the mother cell of these new born neurons can be described as Sox2^+^, therefore also as NeuN^−^, as these two markers exclude each other within the IGL of the adult cerebellum (Supplementary Figure 1a–c).

Since its initial discovery in 1962 the existence of adult neurogenesis has now been widely accepted and great progress has been made in the understanding of the process. Our data further improve this notion by identifying a so far unknown neurogenic niche in the mature cerebellum of adult mice. Further investigation will be necessary to understand and to take advantage of this finding.

## Material and Methods

All experiments and methods, including those using human tissue samples, were carried out in accordance with relevant guidelines and regulations. All mouse experiments were approved by the local government of Upper Bavaria (Regierung von Oberbayern). Human samples were used in accordance with the guidelines and regulations of the local ethics committee. All patients had given their informed consent; all tissue samples were irreversibly anonymized.

### Transgenic mice

*Sox2-creER* ^*T2*^ mice^9^, *hGFAP-cre* mice^10^ and *tdTomato* reporter mice^11^ were obtained from the Jackson Laboratory. *Nestin-GFP* mice were used as previously described^12^. Genotyping was performed by PCR analysis using genomic DNA from ear biopsies. Primers for *Cre* ^13^ and *tdTomato* ^14^ have been published previously.

### Human samples

Human samples were formalin-fixed and paraffin-embedded. Three cerebella without any clinical or neuropathological changes and three cerebella with infarcts in the cerebellum were immunohistochemically stained and evaluated.

### Tamoxifen-induction and BrdU pulse chase

Cre-ER^T2^ -dependent recombination *in vivo* induction of cre activity was achieved by intraperitoneal tamoxifen (Sigma-Aldrich) injection. Animals were injected with tamoxifen at 50 mg kg^−1^ body weight in corn oil (Sigma-Aldrich). BrdU was also injected intraperitoneally at 50 mg kg^−1^ in saline solution. We established an injection protocol, where we start the course of tamoxifen injections one day before starting the BrdU injections to account for slower kinetics of Tamoxifen until it induces gene expression, while BrdU is incorporated into dividing cells within 2–4 h. Mice were injected with Tamoxifen for 8 and with BrdU for 7 consecutive days and sacrificed after 2 additional days (Supplementary Figure 2a) for all analyses apart from cFos stainings, where mice were injected with Tamoxifen for 8 and BrdU for 7 consecutive days and sacrificed after 5 additional days and a final hour in the enriched environment setting (Supplementary Figure 2b).

### RotaRod/Enriched environment

Adult *Sox2-creER* ^*T2*^ *::tdTomato*-mice (>60 days *postpartum*) were either trained on the RotaRod system (RotaRod advanced; TSE Systems, Bad Homburg, Germany) or placed in an enriched environment (EE) to study reactivity of progenitors and neurogenesis. The experimental scheme of the physical exercise paradigms is shown in Supplementary Figure 2. On the RotaRod, mice were trained twice daily for seven days, one run in the morning, one in the afternoon, after having received daily BrdU and tamoxifen injections prior to the first run. Each mouse had to complete 3 runs per session for at least 10 s (exclusion criterion) and maximally 300 s. Progress in running times was monitored with the TSE RotaRod software. Enriched environment was performed as follows: Mice were placed in a large cage (cage size 42 × 23 × 19 cm) for 7 days with daily injections of BrdU and tamoxifen. The cage contained at least one running wheel, tunnels, ladders and shelter. These settings were changed every two days. For both experimental paradigms, control mice only received BrdU and tamoxifen injections and were then placed back into standard cages. Data from behavioural experiments were obtained from three separate litters that were trained in independent experimental rounds as soon as they were old enough.

### Immunohistochemistry

Paraffin-embedded tissue was sectioned, deparaffinised, and rehydrated before heat-induced antigen retrieval was conducted at 100 °C for 20 min in 10 mM sodium citrate buffer (pH 6) for all antibodies. Immunohistochemical staining was done using primary antibodies (Sox2: 1:200, Abcam ab97959; GFP: 1:200, Santa Cruz sc-8334) and the HRP/DAB Staining System (DAKO) according to the manufacturer’s specifications. Hemalaun was used for nuclear counterstaining. All histological photomicrographs were taken digitally using an Olympus BX50 microscope in combination with the Color View Soft imaging system. For confocal imaging, brains were cut into 50 µm thick sections on a vibratome and stained free-floating. For standard microscopy, 4 µm thin sections were stained on slides. For immunofluorescent double- and triple-staining, sections were washed twice with 1% Triton X-100/PBS and then incubated in blocking buffer (10% normal goat serum in 1% Triton X-100/PBS) for 1 h. For BrdU staining, sections were additionally incubated in 2 N HCl and 0.1 M boric acid buffer (pH 8.5) for 10 min each prior to blocking. Primary antibodies (BrdU: 1:200, abcam ab6326; Blbp^15^: 1:5000; cFos: 1:200, Invitrogen 38–4950; Cre: 1:100, Covance PRB106C; GFP: 1:200, Santa Cruz sc-8334: 1:200; molecular probes A21311: 1:200; Ki67: 1:200, abcam ab16667; NeuN: 1:200, abcam ab128886 or ab104224; Olig2: 1:200, Millipore AB960; Parvalbumin: 1:200, Millipore AB15736; Pax2: 1:200, Zymed 71-6000; RFP: 1:200, antibodies online A234; S100: 1:2000, DAKO 4C4.9 or 1:200 abcam ab14849; Sox2: 1:200, abcam ab97959 or ab79351) were diluted in blocking buffer and applied over night at 4 °C. Next, tissue was washed twice with 1% Triton X-100/PBS and incubated for 1 h with a 1:500 dilution of Alexa-fluorophore conjugated secondary antibodies (goat anti-mouse Alexa488/Alexa555/Alexa647; goat anti-rabbit Alexa488/Alexa555/Alexa647, goat anti-rat Alexa647; Invitrogen) in blocking buffer. Sections were washed twice with PBS, counterstained with 4′,6-diamidino-2-phenylindole (DAPI), and mounted in Fluorescent Mounting Medium (DAKO). All fluorescent images of tissue samples were obtained either on the Zeiss ApoTome.2 structured illumination system in combination with the Zen Blue software (Zeiss, 2012), the Zeiss LSM 780 in combination with the Zen Black software (Zeiss, 2011) or on an Olympus IX50 microscope in combination with the Color View Soft imaging system. All of the stains were repeated at least 3 times on tissue from the same sample and also in at least 3 independent subjects.

All images shown in the figures are representative and have been artificially coloured to colour-safe combinations using Image J.

### Quantification and Statistical analysis

Results were analysed using the Prism5 software (GraphPad). P-values < 0.05 were considered significant. The Mann–Whitney-U test was used to compare the median of 2 groups. Standard t-testing was performed to compare groups with a Gaussian distribution of data according to the central limit theorem. For behavioural experiments quantifications were performed blinded, meaning the investigator did not know whether the tissue analysed came from animals that had or had not undergone RotaRoad training or had or had not been housed in an enriched environment. For other evaluations and quantifications, the investigator did not know which color represented which marker. Samples sizes are given in the appropriate figures where statistical analyses were performed. For qualitative and descriptive figures, we show representative images and analysed brain sections from at least five different animals and counted 300 cells per sections. For quantification of BrdU^+^ RFP^+^ NeuN^+^ cells and Sox^+^ S100^−^ cells in *Sox2-creER* ^*T2*^ *::tdTomato* mice we analysed at least 6 entire midsagittal sections per mouse. To calculate the fraction of Sox2^+^ S100^−^ cells, at least 500 cells were counted for each human sample (control and cerebellar infarct). Results were analysed using the Fisher exact test. All respective histograms illustrate the standard error of the mean (SEM; error bars).

## Electronic supplementary material

Supplementary Figures

Supplementary Video A: Z-stack of progeny from Sox2+ precursor cells in the adult cerebellar cortex

Supplementary Video B: Z-stack of progeny from Sox2+ precursor cells in the adult cerebellar cortex [RFP and DAPI].

Supplementary Video C: Z-stack of progeny from Sox2+ precursor cells in the adult cerebellar cortex [BrdU and DAPI].

Supplementary Video D: Z-stack of progeny from Sox2+ precursor cells in the adult cerebellar cortex [NeuN and DAPI].
