# Supplementary material for: Neurogenesis from Sox2 expressing cells in the adult cerebellar cortex
Source: Sci Rep. 2017 Jul 21;7:6137. doi: 10.1038/s41598-017-06150-x (PMC5522437; doi:10.1038/s41598-017-06150-x)
Supplement: Supplementary file 1 — Supplementary Figures [file 41598_2017_6150_MOESM1_ESM.pdf]

Title

Neurogenesis from Sox2 expressing cells in the adult cerebellar cortex

Authors

Julia Ahlfeld[1, 2], Severin Filser[3, 4], Felix Schmidt[1], Annika K. Wefers[5], Daniel J. Merk[6, 7], Rainer Glaß[8], Jochen Herms[2, 4] and Ulrich Schüller[1, 9, 10, 11]

[1] Center for Neuropathology, Ludwig-Maximilians-University, Munich, Germany

[2] Division of Clinical Pharmacology, Department of Internal Medicine IV, Ludwig-Maximilians-University, Munich, Germany

[3] German Center for Neurodegenerative Diseases (DZNE), Department for Translational Brain Research, Munich, Germany

[4] Munich Cluster of Systems Neurology (SyNergy), Ludwig-Maximilians-University, Munich, Germany

[5] Division of Neuropathology, Heidelberg University Hospital, Heidelberg, Germany

[6] Cancer Biology and Pediatric Oncology, Dana-Farber Cancer Institute, Boston, Massachusetts

[7] Neurobiology, Harvard Medical School, Boston, Massachusetts

[8] Department of Neurosurgery, Ludwig-Maximilians-University, Munich, Germany

[9] Institute of Neuropathology, University Medical Center, Hamburg-Eppendorf, Germany

[10] Research Institute Children's Cancer Center, Hamburg, Germany

[11] Department of Pediatric Hematology and Oncology, University Medical Center, Hamburg-Eppendorf, Germany

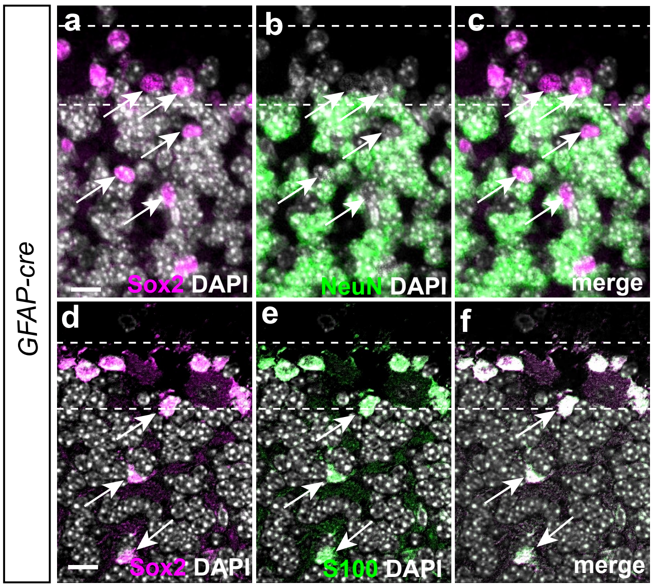

Supplementary Figure 1: Sox2 labels differentiated glia, but no granule cells in the adult cerebellar cortex.

Staining of Sox2 and neuronal marker NeuN shows no colocalization of the two, neither within the PCL nor within the IGL (arrows in a - c). Sox2 expressing cells within the IGL are of glial lineage as shown by colocalization of Sox2 with glial marker S100 (d – f). White lines show perimeter of Purkinje cell layer. Scale bars equate to 30 µm.

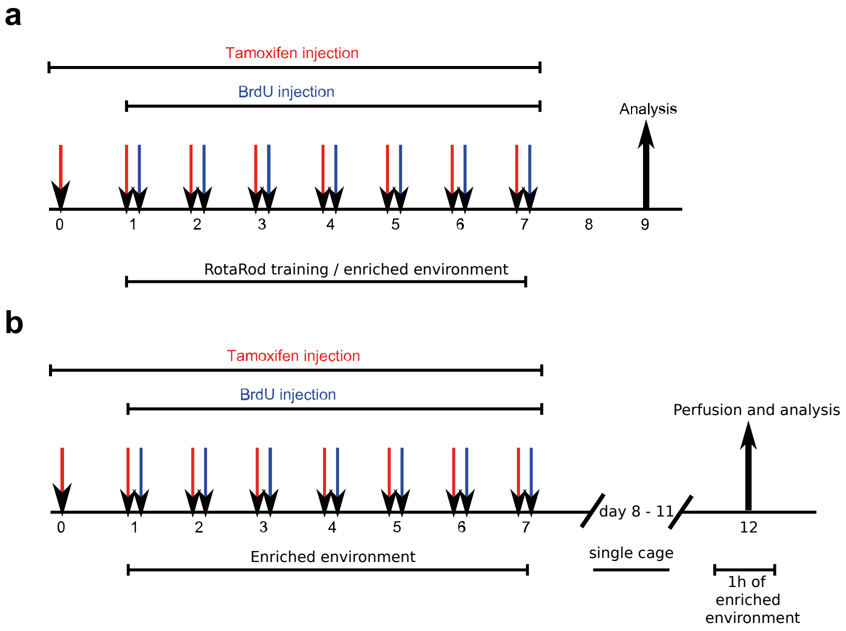

Supplementary Figure 2: Protocols for birth-dating and fate-mapping experiments. Mice were injected with Tamoxifen and BrdU over a time of 8 and 7 days, respectively, according to the schemes above. Mice were analyzed for adult neurogenesis after one course of injections and training (a). For analysis of synaptic integration of new born neurons, mice were kept in an enriched environment for 7 days with daily Tamoxifen and BrdU injections. Mice were then transferred to single cages for 5 days. For induction of cFos expression, mice were exposed to an enriched environment for an hour before perfusion (b).

Supplementary Video A: Z-stack of progeny from Sox2$^+$ precursor cells in the adult cerebellar cortex. Combined BrdU birth-dating and fate-mapping experiments show RFP$^+$ progeny from Sox2$^+$ cells (magenta) that had incorporated BrdU during the observation period (cyan) and had also acquired neuronal differentiation as shown by staining for the granule cell marker NeuN (green). Each individual marker is also shown with the corresponding DAPI channel alone. The confocal images were taken after a 7/8-day treatment with BrdU and Tamoxifen respectively and another 2 days before sacrifice. To create the z-stack, 34 confocal planes were acquired every 0.4 µm.

Supplementary Video B: Z-stack of progeny from Sox2$^+$ precursor cells in the adult cerebellar cortex [RFP and DAPI]. RFP$^+$ progeny from a Sox2$^+$ cell (magenta; DAPI - grey) that had arisen during an 8-day course of Tamoxifen treatment and another 2 days before sacrifice. To create the z-stack, 34 confocal planes were acquired every 0.4 µm.

Supplementary Video C: Z-stack of progeny from Sox2$^+$ precursor cells in the adult cerebellar cortex [BrdU and DAPI]. BrdU birth-dating shows a cell within the mature IGL that had incorporated BrdU (cyan; DAPI - grey) during the observation period of a 7-day treatment course with BrdU and another 2 days before sacrifice. To create the z-stack, 34 confocal planes were acquired every 0.4 µm.

Supplementary Video D: Z-stack of progeny from Sox2$^+$ precursor cells in the adult cerebellar cortex [NeuN and DAPI]. NeuN-positive granule neurons of the mature IGL (green; DAPI - grey). The confocal images were taken after a 7/8-day treatment with BrdU and Tamoxifen respectively. To create the z-stack, 34 confocal planes were acquired every 0.4 µm.
